# How machine learning on real world clinical data improves adverse event recording for endoscopy

**DOI:** 10.1038/s41746-025-01826-5

**Published:** 2025-07-10

**Authors:** Stefan Wittlinger, Isabella C. Wiest, Mahboubeh Jannesari Ladani, Jakob Nikolas Kather, Matthias P. Ebert, Fabian Siegel, Sebastian Belle

**Affiliations:** 1 Department of Medicine II, University Medical Center Mannheim, Medical Faculty Mannheim, Heidelberg University, Mannheim, Germany; 2 Else Kroener Fresenius Center for Digital Health, Faculty of Medicine and University Hospital Carl Gustav Carus, TUD Dresden University of Technology, Dresden, Germany; 3 Department of Biomedical Informatics, Mannheim Institute for intelligent Systems in Medicine (MIISM), Medical Faculty Mannheim, Heidelberg University, Mannheim, Germany; 4 Department of Medicine I, Faculty of Medicine and University Hospital Carl Gustav Carus, TUD Dresden University of Technology, Dresden, Germany; 5 Medical Oncology, National Center for Tumor Diseases (NCT), University Hospital Heidelberg, Heidelberg, Germany, Heidelberg, Germany; 6 DKFZ Hector Cancer Institute at the University Medical Center, Mannheim, Germany; 7 Molecular Medicine Partnership Unit, European Molecular Biology Laboratory, Heidelberg, Germany

**Keywords:** Health care, Medical research

## Abstract

Endoscopic interventions are essential for diagnosing and treating gastrointestinal conditions. Accurate and comprehensive documentation is crucial for enhancing patient safety and optimizing clinical outcomes; however, adverse events remain underreported. This study evaluates a machine learning-based approach for systematically detecting endoscopic adverse events from real-world clinical metadata, including structured hospital data such as ICD-codes and procedure timings. Using a random forest classifier detecting adverse events perforation, bleeding, and readmission, we analysed 2490 inpatient cases, achieving significant improvements over baseline prediction accuracy. The model achieved AUC-ROC/AUC-PR values of 0.9/0.69 for perforation, 0.84/0.64 for bleeding, and 0.96/0.9 for readmissions. Results highlight the importance of multiple metadata features for robust predictions. This semi-automated method offers a privacy-preserving tool for identifying documentation discrepancies and enhancing quality control. By integrating metadata analysis, this approach supports better clinical decision-making, quality improvement initiatives, and resource allocation while reducing the risk of missed adverse events in endoscopy.

## Introduction

Endoscopic interventions have become critical tools in diagnosing and treating various gastrointestinal (GI) diseases, offering minimally invasive solutions with reduced patient recovery time and morbidity. However, as with any medical procedure, these interventions carry inherent risks of adverse events, which may range from minor adverse events to life-threatening situations^1^. Despite advancements in technology and technique, the occurrence of adverse events remains a significant concern, necessitating a thorough understanding of their nature, frequency, and contributing factors.

Accurate and consistent documentation of adverse events is essential to enhancing the overall quality and safety of endoscopic procedures. Not only does comprehensive documentation provide valuable data for individual patient care and informed clinical decision-making, but it also facilitates broader quality improvement efforts^2^. By systematically recording adverse events, healthcare providers can identify patterns, benchmark performance, and implement targeted interventions to reduce risk. Additionally, such data is critical for developing evidence-based guidelines, conducting risk assessments, and improving the training of endoscopists.

Adverse events at the University Hospital Mannheim (see Supplementary Table 1) and in the German screening colonoscopy registry are underreported^3^. Systematic recording of adverse events is challenging because they can arise at any point in a patient’s medical history. While such recordings may work reasonably well for adverse events identified during the endoscopy procedure itself, they fail to capture those that emerge later, either during the subsequent hospital stay or upon readmission. In some instances, readmission may occur at a different hospital, further complicating documentation. Consequently, these adverse events may remain undocumented in the systematic recording system, even though they might be noted in other written records, such as discharge letters.

In medicine, machine learning has been widely applied to computer vision^4,5^, one prominent example being applications within endoscopy^6^. It has also been used to predict the onset of diseases^7–11^ from clinical data. Large language models (LLM), a subset of machine learning, have demonstrated their utility in systematically extracting information from unstructured electronic health records^12–14^. LLMs have also been utilized to extract information from the Mannheim colonoscopy dataset^15^ used in this work.

**Fig. 1:**
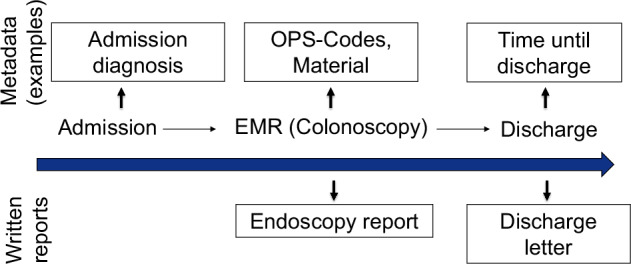
Example of data generated during a hospital stay. This figure displays an example of data generated during a hospital stay, which includes both unstructured data, primarily in the form of text (e.g., endoscopy reports and discharge letters), and structured data (metadata), such as diagnoses, materials used during endoscopy, and time until discharge. For a comprehensive list of the metadata used, refer to Supplementary Tables 2–3.

In this paper, we investigate the potential of detecting adverse events from metadata, that is, structured data such as hospital stay duration, material used during endoscopy, ICD codes, and other related information (see Fig. 1 for examples and Supplementary Tables 2–3 for full list)^16^. Crucially, our objective is to detect adverse events that have already taken place, not to predict their occurrence in advance. Detecting adverse events from metadata could enhance the accuracy of adverse event records and help identify discrepancies between written reports and their corresponding metadata. Specifically, we aim to investigate whether certain adverse events leave identifiable signatures within the metadata that can be extracted using machine learning.

## Results

We focus on three key adverse events associated with endoscopic mucosal resection (EMR): bleeding, perforation, and readmission within 30 days due to EMR-related issues (see supplementary material for consensus definitions). If bleeding or perforation became apparent during or after readmission, it was classified as readmission rather than as bleeding or perforation, respectively.

We trained a machine learning algorithm, more specifically a random forest classifier, on the metadata (see Supplementary Tables 2–3 for features used). To test our method, we utilized 2490 cases of inpatient stays including at least one endoscopic procedure with endoscopic mucosal intervention performed between 2010 and 2022 at the University Hospital Mannheim. A general characterization of the cohort is provided in Table 1. Cohort characteristics of the training dataset are shown in Table 2, and those of the test dataset are presented in Table 3. Additional details of the cohort characteristics can be found in Supplementary Tables 4–7. No restrictions were applied regarding the reason for hospitalization. The most common diagnoses were polyps and benign neoplasms in colon, cecum, or rectum.

**Table 1:** Cohort characteristics of the dataset

Category	Number of entries	Median value	Average value	Standard deviation
Age in years	2490	67.8	66.7	11.8
Charlson comorbidity index	2490	0	1.1	2.0
Barthel index	477	100	89.1	21.0
Number of blood transfusions	2490	0	0.06	0.31
Height in cm	10	178.5	177	8.0
Weight in kg	12	78.5	64.8	33.0
Patient Clinical Complexity Level	2490	0	0.75	1.26
Number of procedures during hospital stay	2490	1	1.01	0.06
Duration of procedure in min	2490	57.0	61.7	29.0
Admission-to-discharge time in days	2490	2.1	4.5	8.00
Procedure-to-discharge time in days	2483	1.9	3.0	5.2

For the given cohort, the median, average value, and corresponding standard deviation of selected features are displayed. The number of entries may be smaller than the total number of available cases (2490), as certain features may not be available for all cases.

**Table 2:** Cohort characteristics of the training dataset

Category	Number of entries	Median value	Average value	Standard deviation
Age in years	1990	67.6	66.6	11.8
Charlson comorbidity index	1990	0	1.1	2
Barthel index	461	100	89	21
Number of blood transfusions	1990	0	0.06	0.3
Height in cm	10	178.5	177	8
Weight in kg	12	78.5	64.84167	32.99244
Patient Clinical Complexity Level	1990	0	0.7	1.2
Number of procedures during hospital stay	1990	1	1.002	0.04
Duration of procedure in minutes	1990	56.7	61.6	29.6
Admission-to-discharge time in days	1990	2.1	4.4	7.6
Procedure-to-discharge time in days	1986	1.9	2.9	4.5

The cohort characteristics of the training dataset (1990 cases) are presented as median, mean, and standard deviation values.

**Table 3:** Cohort characteristics of the test dataset

Category	Number of entries	Median value	Average value	Standard Deviation
Age in years	500	68.4	66.7	11.8
Charlson comorbidity index	500	0	1.2	2.1
Barthel index	16	87.5	84.4	18
Number of blood transfusions	500	0	0.06	0.39
Height in cm	0	-	-	-
Weight in kg	0	-	-	-
Patient Clinical Complexity Level	500	0	0.94	1.4
Number of procedures during hospital stay	500	1	1.01	0.09
Duration of procedure in minutes	500	57	61.7	29
Admission-to-discharge time in days	500	2.1	4.9	9.5
Procedure-to-discharge time in days	497	1.9	3.4	7.2

The cohort characteristics of the test dataset (500 cases) are summarized using the median, mean, and standard deviation. For height and weight, no entries were available; therefore, their median, mean, and standard deviation are not reported.

**Fig. 2:**
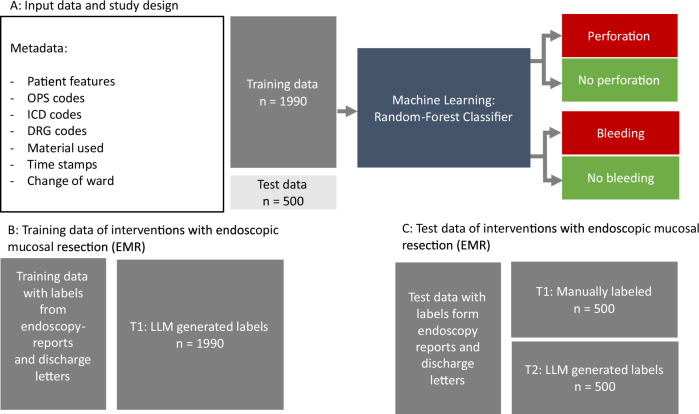
Training and testing scheme for adverse events perforation, and bleeding. For adverse events and perforation adverse events, the scheme for training and testing is displayed. For this purpose a combination of LLM-generated and manually generated labels was used. The random forest was trained for two types of adverse events, perforation and bleeding using a training set with *n* = 1990 cases. The labels for the training set were obtained by running a large language model on the endoscopy reports and discharge letters. The performance metrics were obtained by testing on the remaining *n* = 500 manually labeled cases representing the ground truth. To estimate the stability of the machine learning algorithm, the large language model labels were used for the entire data set (*n* = 2490). With these, we performed random subsampling with 100 iterations. In each iteration, the data was randomly split into training (*n* = 1990) and test set (*n* = 500). From this, the standard deviation of the performance metrics was calculated. Perforation or bleeding that occurred after readmission was not classified as adverse events, perforation or bleeding, but rather as adverse event readmission. The listed data is available at discharge, allowing the detection of adverse events such as bleeding or perforation to be performed at discharge or any later time.

**Fig. 3:**
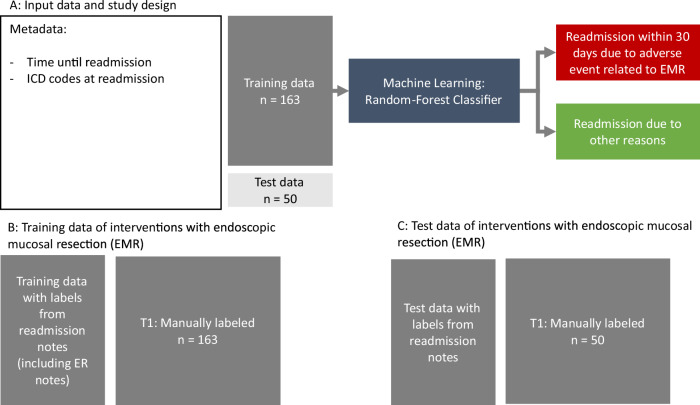
Training and testing scheme for adverse events of readmission. Training and testing scheme for adverse event readmission within 30 days due to adverse events in connection with previously performed EMR. The entire data set, *n* = 213, consisting of all readmissions within 30 days was manually labeled. Given the limited sample size, the metadata used was restricted to the time until readmission and the ICD codes recorded at readmission. The random forest classifier was trained on *n* = 163 cases and tested on *n* = 50 cases. To evaluate the stability of the machine learning algorithm, random subsampling was performed over 100 iterations, with different splits between training and testing sets in each iteration. The listed data is available at readmission, allowing the detection of adverse event readmission to be performed at readmission or any later time.

All 2490 cases were utilized for classifying the adverse event types of bleeding and perforation. For readmission as an adverse event, 213 cases were analyzed; these represent instances where patients were readmitted within 30 days post-discharge. The study design for the adverse events perforation and bleeding is illustrated in Fig. 2, while the scheme for readmission as an adverse event is presented in Fig. 3.

For the supervised machine learning algorithm, labels were obtained by utilizing the written reports (endoscopy reports, discharge notes, readmission notes). These were either reviewed by an expert (referred to as “manually generated labels”) or generated using a large language model (LLM), referred to as “LLM-generated labels.” The manually generated labels were generally considered the ground truth, meaning they were assumed to be fundamentally correct. Among the 500 manually labeled cases used for testing adverse events, bleeding and perforation, 134 cases involved bleeding, and 37 cases involved perforation as an adverse event. Of the 213 manually labeled cases available for readmission analysis, 45 were identified as adverse event readmissions.

### Model achieves high classification performance for readmission

The results were evaluated using receiver operating characteristics (ROC) and precision-recall (PR) curves. The primary performance metrics considered were the area under the curve (AUC) for both. Due to class imbalance, the precision-recall AUC (AUC-PR) was prioritized as the main performance metric over the ROC AUC (AUC-ROC)^17^. The AUC-PR performance of our machine learning algorithm was compared against a baseline dummy classifier that performs random classification.

**Fig. 4:**
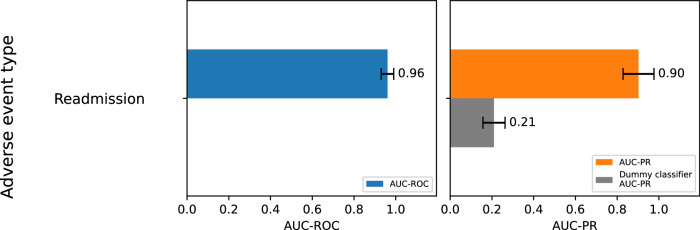
Test results for adverse event readmission. Test results (AUC-ROC and AUC-PR) and errors for adverse event readmission within 30 days due to adverse events in connection with previously performed EMR are displayed. The dataset (*n* = 213) with manually labeled data was randomly split into a training set (*n* = 163) and a testing set (*n* = 50). This random subsampling process was repeated 100 times. The AUC-ROC and AUC-PR values were calculated as the mean across all runs, with error bars representing the standard deviation.

**Fig. 5:**
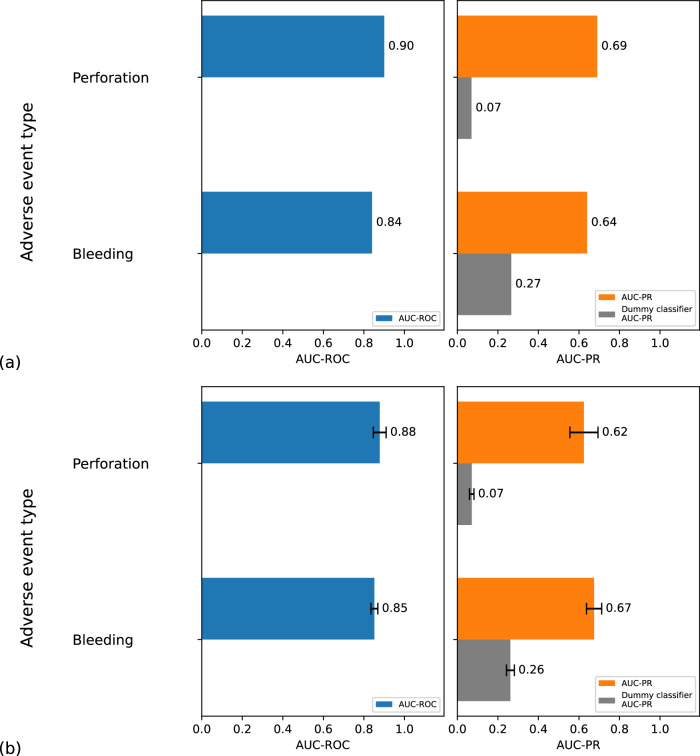
Test results for adverse events bleeding and perforation. **a** The test results for adverse events bleeding and perforation (AUC-ROC and AUC-PR) are displayed. The model was trained on a training set (*n* = 1990) with labels generated by a large language model and tested on a manually labeled test set (*n* = 500). Direct error bars cannot be computed for this process, as random subsampling would require manual labels for all cases. **b** Estimated error values using only labels generated by a large language model are shown. Labels generated by a large language model are used for both training (*n* = 1990) and testing (*n* = 500). This process is repeated over 100 iterations using random subsampling, with a different split of training and test data in each iteration. Performance metrics (AUC-ROC, AUC-PR, and dummy classifier) are calculated as mean values, with the error bars representing the standard deviations shown in the plot.

The results of our analysis are presented in Figs. 4 and 5. The AUC-PR was calculated to evaluate the accurate classification of adverse events, yielding values of 0.69 for perforation (compared to a dummy classifier’s 0.07), 0.64 for bleeding (dummy classifier: 0.27), and 0.9 for readmission (dummy classifier: 0.21). The corresponding AUC-ROC values are 0.9 for perforation, 0.84 for bleeding, and 0.96 for readmissions. The confusion matrices and individual AUC-ROC/-PR curves are provided in Supplementary Figs. 1–6. For comparison, two gradient-boosted decision tree algorithms (LightGBM and CatBoost) and a deep neural network (TabNet) were applied to the same dataset. CatBoost performed on par, achieving AUC-ROC scores of 0.90 for perforation, 0.85 for bleeding, and 0.95 for readmission, as well as AUC-PR scores of 0.71 for perforation, 0.65 for bleeding, and 0.89 for readmission. For more details, see Supplementary Fig. 7.

The ROC and PR curves shown in Supplementary Figs. 4–6 show that perforation and readmission achieve a perfect positive predictive value (PPV) of 1 at small sensitivity (true positive rate) values. In contrast, bleeding never reaches a PPV of 1, even at low sensitivity values.

### Cross-validation demonstrates stable performance

To assess the stability of the random forest classifier, we employed random subsampling as a cross-validation technique^18^. The error bars, which reflect the variability of the performance estimates, are shown in Fig. 4 for adverse event readmission and in Fig. 5b for adverse events bleeding and perforation. Note that, for perforation and bleeding, random subsampling could not be performed on the actual test data, as only 500 manually labeled cases were available. Instead, this analysis was conducted with labels generated by a large language model (2490 cases in total) for the entire dataset. The AUC-ROC and AUC-PR values in Fig. 5b are approximately within 10% of those in Fig. 5a, where manually labeled cases were used as test data. The error bars here serve as an estimation of the variability that would exist if all labels were manually generated.

As an additional validation step (see Supplementary Table 8), we analyzed the subset of 500 samples for which both manual and LLM-generated labels were available, performing 1000 bootstrapping iterations to assess model performance for adverse events perforation and bleeding. The results showed similar AUC-ROC and AUC-PR values for both label types, potentially indicating comparable model performance when using either LLM-generated or manual labels.

### Metadata deviations from regular care plans strongly indicate adverse events

**Fig. 6:**
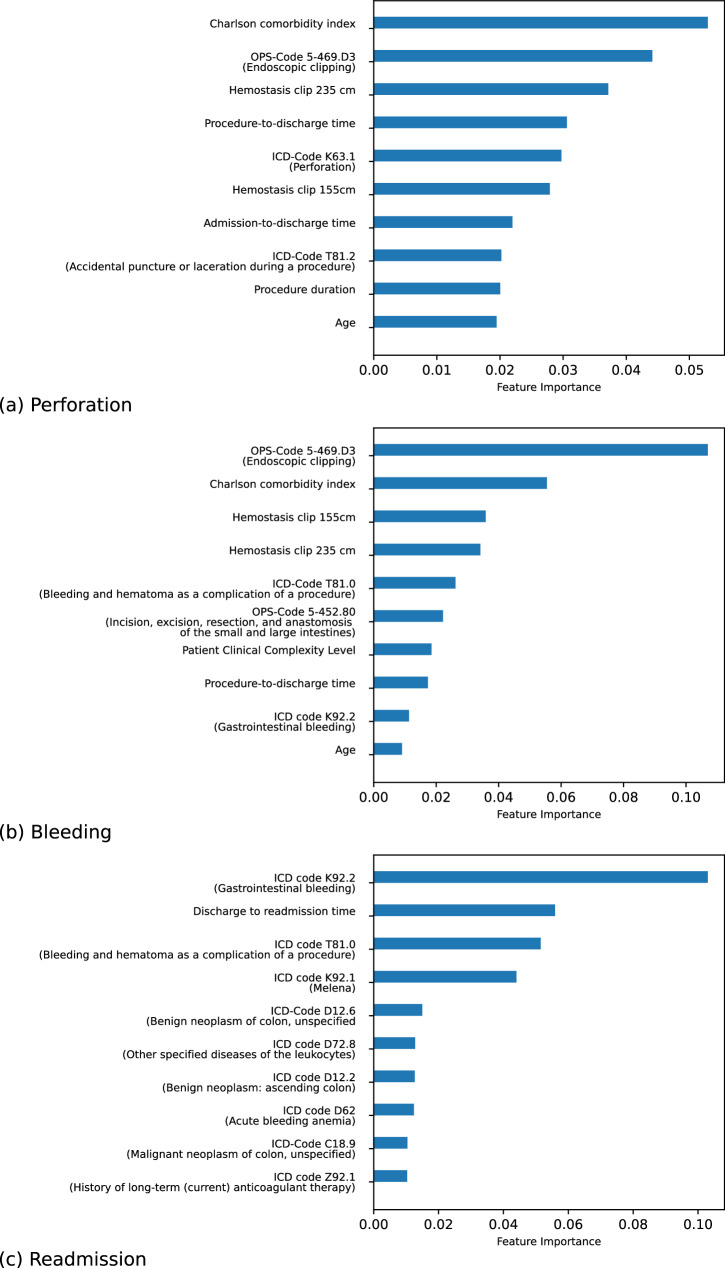
Ten most important features for adverse events perforation, bleeding, and readmission. The 10 most important features for **a** perforation **b** bleeding and **c** readmission are displayed. SHAP was used to determine feature importance.

For perforation, the top three key features were “Charlson comorbidity index”, “OPS-Code 5-469.D3 (endoscopic clipping),” and “hemostasis clipping 235 mm”. The most important features, as determined by SHAP^19^, are displayed in Fig. 6. For bleeding, the top three features were “OPS-Code 5.493.D3 (endoscopic clipping)”, “Charlson comorbidity index,” and “hemostasis clipping 155 cm”. In both cases, the Charlson comorbidity index and the use of clips were associated with adverse events. These deviations from the normal care plan suggest that an adverse event may have occurred. Interestingly, while the ICD-code K63.1 (perforation) might seem like a strong indicator of perforation, in our test set, only 8 out of 37 cases with this code were actually related to EMR-induced perforations, therefore requiring additional features to achieve robust classification performance.

For readmission, the top three predictors are “ICD-code K92.2 (gastrointestinal bleeding)”, “Discharge to readmission time,” and “ICD-code T81.0 (bleeding and hematoma as a complication of a procedure)”. This suggests that rapid readmission accompanied by bleeding—whether related to a procedure or occurring in the gastrointestinal tract—may indicate an EMR-related adverse event.

### Sequential feature selection reveals performance declines with reduced feature sets

To evaluate whether a reduced number of features could achieve similar performance, experiments were conducted using only the top one, two, or three features for each type of adverse event. For example, it was hypothesized that ICD code K63.1 (perforation) might alone provide superior predictive power for perforation. However, the results showed that reducing the number of variables caused noticeable declines in performance, as measured by AUC-ROC and AUC-PR. The decline in performance was particularly pronounced for perforation and bleeding, while for readmission, the decline is less pronounced. The detailed results are shown in Supplementary Fig. 8.

## Discussion and conclusion

This work demonstrates the potential of leveraging metadata to improve the systematic documentation of adverse events associated with endoscopic interventions. By applying machine learning methods, specifically a random forest classifier, to analyze hospital metadata, we identified patterns associated with specific adverse events, perforation, bleeding, and readmission, which offers the potential of automating and enhancing the accuracy of records of adverse events. Our machine learning algorithm demonstrated a substantial improvement over the baseline.

A limitation of this study is that the model was developed and evaluated using data from a single hospital. While we deliberately selected generic features that should generalize well, external validation with data from other institutions would be necessary to confirm the robustness and generalizability of our approach to different settings. Patient populations and the types of interventions performed may vary depending on the level and type of care, for example, between a tertiary care institution such as the University Medical Center Mannheim, other hospitals or clinics, and outpatient colonoscopy settings. External validation could help ensure that the observed patterns are not specific to our dataset and that the model performs consistently across various healthcare environments. Importantly, generalizability can only be robustly demonstrated when large-scale, representative data from diverse medical institutions are available—an undertaking that is not feasible at the current stage. Nevertheless, our method offers substantial practical value: it can be rapidly implemented across clinical settings with minimal human effort. The low manual overhead ensures that individual centers can adopt the system efficiently, requiring only modest local adaptation. We view this ease of deployment as a central strength and a key contribution of our work. Another limitation of our study is that potential readmissions to other hospitals are not captured in our dataset. However, with the electronic patient record system now being made available in Germany, this limitation may be mitigated in the future.

An important finding of this study is that for adverse events perforation and bleeding, no single feature, or even a small subset of features, can solely explain the model’s predictions. For the adverse event of readmission, three features already yield respectable classification performance, but adding more features further improves performance. The results indicate that the prediction of any adverse event type relies on the complex interplay of multiple features in the metadata. This underscores the need for a comprehensive approach in predictive modeling, where the combination of variables, rather than individual ones, leads to optimal performance.

Our model was particularly successful in detecting adverse events related to perforation and readmission, yielding strong performance metrics. Adverse events of the type bleeding were also detectable, although with somewhat lower predictive accuracy. This may be due to the less distinct and consistent signature of bleeding in the metadata, which complicates precise prediction. Unlike perforation, which is often linked to specific indicators such as increased use of clipping material, longer hospital stays, and even specific ICD codes, bleeding appears to lack such clear markers. This could be because bleeding does not significantly alter the course of therapy in a way that is easily captured in metadata, making it harder to detect or track compared to perforation, which has more direct and identifiable association with clinical data. Given that perforation and readmission cause higher costs to the health system, and these adverse events can be detected more accurately, the proposed system might be particularly effective in identifying costly and resource-intensive adverse events.

Even when trained on limited, noisy datasets, such as in this study, the model demonstrated its robustness and reliability, evidenced by narrow error bars for the AUC-ROC and AUC-PR. This indicates that the algorithm performs consistently across different subsets of the data and is not overly sensitive to small variations. Overall, these findings suggest that our machine learning approach not only enhances the predictive accuracy for rare adverse events but also maintains stability across various data subsets. This makes it a promising tool for clinical decision-making. The lower accuracy for bleeding, however, highlights an area for further refinement, where additional data, feature engineering, or potentially a narrower definition of bleeding might improve its detection. In the future, incorporating additional data could potentially enhance the accuracy of classifying any of the investigated adverse events.

Furthermore, if the tool has already been trained and is solely deployed, it operates independently of written text. This enables metadata analysis to be conducted in a privacy-preserving manner, avoiding the need to transfer patient-identifying data to cloud servers, an approach not always feasible with large language models. Furthermore, when trained on high-quality labeled data and deployed in real-world clinical settings, our method has the potential to flag adverse events that may not be explicitly documented in free text but are discernible through patterns in structured data.

Enhancing the integration between metadata analysis and patient records has the potential to significantly improve the accuracy of adverse event documentation, while reducing inconsistencies between reported and undocumented events^20,21^. Regarding individual patients, if information about an adverse event is lost, it could potentially compromise their subsequent treatment and care. Regarding the improvement of treatment outcomes now and in the future, a complete and accurate systematic record of all adverse events is essential for learning and refining clinical practices.

Among others, we envision the following practical implementation within a comprehensive clinical support system for the electronic health record: at discharge, our machine learning algorithm could check whether any adverse events have occurred based on the structured metadata. This could then be compared to what is written in the discharge letter. If a discrepancy is detected, an alert could prompt clinicians to verify whether all significant events have been accurately documented. For example, an adverse event may have occurred and been recorded as an ICD diagnosis, but due to handover mistakes, it might not be properly documented in the discharge letter, especially if the responsible physician for the colonoscopy differs from the one handling the discharge. Incorrectly coded ICD diagnoses could potentially be identified if they do not match the written reports.

Our study also demonstrated how large language model (LLM)-based text mining could assist in this process by automatically extracting relevant information from unstructured text in clinical documents, such as discharge letters. By leveraging LLMs, the system could identify adverse events and discrepancies between the structured and unstructured data. This capability could be further enhanced to perform automated cross-checking between text-based EHR documents and structured healthcare data, increasing the accuracy and completeness of clinical documentation.

An automated feedback loop could also be envisioned to inform the endoscopist who performed the intervention about the occurrence of an adverse event.

Checking for adverse events could also be conducted at other times, such as at readmission or even during the yearly review of adverse events. Specifically, to improve data quality in the systematic recording of adverse events, this algorithm could flag potential cases retrospectively, identifying occurrences of adverse events that might not have been fully documented.

Challenges for a real world pilot project include usability and interpretability. So far, our algorithm only classifies the adverse event without providing reasoning. Future work could focus on improving interpretability, for instance, by exploring the explainability of specific instances using SHAP, local interpretable model-agnostic explanations^22^, or other techniques. Additionally, user feedback should be integrated to enhance the learning process, which could be done by implementing reinforcement learning algorithms.

## Methods

Ethical approval has been received from Ethics Committee II, Medical Faculty of Mannheim, Heidelberg University (approval number 2021-694). Pseudonymized data processing in retrospective studies is exempt from obtaining individual patient consent under the applicable regulatory framework.

### Labeling process

Labels for the supervised machine learning algorithm were obtained from written reports, including endoscopy reports, discharge notes, and readmission notes. Labels reviewed and assigned by an expert are termed “manually generated labels,” while those produced by a large language model (LLM) are referred to as “LLM-generated labels.” The manually generated labels were considered the ground truth and assumed to be accurate. Specifically, for adverse events bleeding and perforation, the ground truth was established using endoscopy and discharge notes, while adverse events in connection to readmission were identified through readmission notes, in conjunction with previous reports to determine their association with prior endoscopic mucosal resections. In order to obtain reliable performance metrics, testing was only performed on cases with manual labels available.

For readmission, all 213 cases were manually labeled, providing a complete set of high-quality ground truth data. However, for the adverse events bleeding and perforation, only 500 out of the 2490 cases were manually labeled and used as the test dataset. The remaining cases, used as training data, were labeled using the large language model Llama-2 70b in a fine-tuned version for German language (Llama-2 70B “Sauerkraut”) as described in ref. ^15^. The referenced paper provides details of the implementation. A simple prompt asking to extract adverse events was found to work well. The ground truth was established using the definitions provided in Supplementary material.

It is important to note that this method of using large language models may introduce slight inaccuracies due to the potential for noisy labels generated by the model. For an analysis of the quality of these LLM-generated labels, see Supplementary Fig. 9.

### Data preprocessing and feature engineering

Data processing was performed using pandas^23^. The data was delivered through multiple Excel files that were combined using pandas. Unstructured data, such as written texts, was removed from the dataset. One-hot encoding was used for categorial data. For materials used during endoscopy, the quantity of each material was also encoded. For example, if three clips of type “hemostasis clip 235 cm” were used, this was encoded in the “hemostasis clip 235” category as 3.

If appropriate, imputation using the median value was applied. This means that if values were missing for a specific case, they were replaced with the median value. If no readmission took place, feature “admission-to-readmission time” was set to 1000 days. In seven cases, the patient underwent two endoscopic interventions, both including EMR in one hospital stay. In these cases, the DRG, OPS codes, as well as the material used, were combined. The date of the first intervention was used to calculate the feature value “procedure-to-readmission time,” while the maximum value for feature “procedure time” was used. Besides this, the circumstance of multiple interventions in one hospital stay was captured with the feature “number of procedures”.

### Machine learning algorithm

A random forest classifier, implemented in Scikit-learn^24^, was trained for classification. A random forest classifier can be expected to be more robust against overfitting, which is a concern given our small and potentially noisy dataset. Other machine learning algorithms were also tested but did not demonstrate any significant performance improvement. In particular, two gradient-boosted decision tree algorithms (LightGBM^25^ and CatBoost^26^) and one deep learning algorithm optimized for tabular data (TabNet^27^) were applied to the data after completion to facilitate performance evaluation.

Feature selection was conducted using backward feature elimination, an iterative method that begins with all available features and systematically removes the least significant ones. At each step, the features contributing the least to the model’s performance, as determined by an evaluation metric (in this case, impurity-based feature importance), are excluded. This process continues until the desired number of features is reached. Before backward feature selection, a total of 4547 features were available for perforation and bleeding, and 493 features for readmission.

### Hyperparameter tuning and class imbalance

Hyperparameter tuning is the process of selecting the optimal combination of model parameters to maximize model performance. This is typically achieved by dividing the training data into smaller subsets, such as a training subset and a validation subset. The training subset is used to fit the model, while the validation subset evaluates its performance for each set of hyperparameter choices. The space of possible hyperparameter combinations is then systematically explored using a grid search to identify the optimal configuration (other search algorithms, such as Bayesian optimization, could also be considered as potential alternatives).

For adverse events perforation, and bleeding, the number of features was treated as a hyperparameter, ranging from 50 to 500 features in increments of 50. For adverse event readmission, the number of features was set to 100. Hyperparameter tuning of the random forest’s internal parameters was tested but had no noticeable impact on performance, either positive or negative. Consequently, all results were achieved with consistent settings: The number of estimators of the random forest (n_estimators) was set to 1000, all other parameters were set to their default values in SciKit-learn. To address class imbalance, balanced class weights, synthetic data augmentation using SMOTE, and Balanced Random Forests were tested, but did not yield any performance improvements.

### Feature importance and stability

The most important features were identified using SHAP^19^. To evaluate the algorithm’s stability, random subsampling^28^ was conducted 100 times. In each iteration, the data was randomly split into training and test sets, with the machine learning algorithm trained on the training set and evaluated on the test set. For adverse event of readmission, all 213 cases included manual labels, allowing direct random subsampling on the full dataset. However, random subsampling could not be directly applied to adverse events perforation and bleeding due to the limited availability of manual labels (500, defined as ground truth). For these cases, algorithm stability was assessed using labels generated by a large language model applied to the entire dataset, which are considered an approximation.

### Evaluation metrics

As target metrics, we evaluated the area under the curve of the receiver operating characteristic (AUC-ROC) and the area under the precision-recall curve (AUC-PR). In particular, we focused on the precision-recall curve due to the imbalance in our dataset. Given the imbalanced distribution of adverse events and the need to accurately identify adverse events while minimizing false positives, we regard AUC-PR as the most relevant performance metric^29^. In contrast, the AUC-ROC may provide overly optimistic estimates when applied to highly imbalanced datasets^17^.

The AUC-PR is assessed relative to its baseline, which is defined by the performance of a dummy classifier (i.e., one that makes random predictions). For such a dummy classifier, the AUC-PR corresponds to the occurrence rate of adverse events in the test dataset. For instance, if 20 adverse events occur in 100 cases, the AUC-PR for a dummy classifier would be calculated as 20 divided by 100, resulting in a value of 0.2 (note that the AUC-ROC of a dummy classifier is always 0.5, regardless of the rate at which adverse events occur.)

This baseline serves as a meaningful reference point to assess the model’s effectiveness in detecting rare adverse events. The error (defined as the standard deviation) of the area under the curve metrics serves as an estimate of the stability of the machine learning algorithm.

## Supplementary information

Paper_Metadaten_ML_supplementary

## Data Availability

The data are not publicly available because they consist of electronic health records collected at the University Hospital Mannheim. Publicly sharing these data violates the terms of the original ethical approval and could compromise patient privacy. De-identified patient data or other prespecified data will be made available upon approval of a written request and the signing of a data sharing agreement.

## Code availability

The underlying code of this project is available on GitHub (https://github.com/stewitt/MLEndoAE). The analysis was conducted using Python (version 3.10.11) along with the following libraries, scikit-learn (version 1.0.2), NumPy (version 1.24.2), Pandas (version 2.0.0), Matplotlib (version 3.7.1), LightGBM (version 4.6.0), CatBoost (version 1.2.7), PyTorch (version 2.0.1), and SHAP (version 0.46.0).

## References

[1] Kavic, S. M. & Basson, M. D. Complications of endoscopy. *Am. J. Surg.* **181**, 319–332 (2001). 11438266 10.1016/s0002-9610(01)00589-x

[2] Mergener, K. Defining and measuring endoscopic complications: more questions than answers. *Gastrointest. Endosc. Clin. N. Am.* **17**, 1–9 (2007). 17397772 10.1016/j.giec.2007.01.001

[3] Adler, A. et al. Data quality of the German screening colonoscopy registry. *Endoscopy* **45**, 813–818 (2013). 24019130 10.1055/s-0033-1344583

[4] Esteva, A. et al. Deep learning-enabled medical computer vision. *NPJ Digit Med.* **4**, 5 (2021). 33420381 10.1038/s41746-020-00376-2 PMC7794558

[5] Harerimana, G., Kim, J. W., Yoo, H. & Jang, B. Deep learning for electronic health records analytics. *IEEE Access* **7**, 101245–101259 (2019).

[6] Ali, S. Where do we stand in AI for endoscopic image analysis? Deciphering gaps and future directions. *NPJ Digit Med.* **5**, 184 (2022). 36539473 10.1038/s41746-022-00733-3 PMC9767933

[7] Tang, A. S. et al. Leveraging electronic health records and knowledge networks for Alzheimer’s disease prediction and sex-specific biological insights. *Nat. Aging* **4**, 379–395 (2024). 38383858 10.1038/s43587-024-00573-8 PMC10950787

[8] Ravaut, M. et al. Predicting adverse outcomes due to diabetes complications with machine learning using administrative health data. *NPJ Digit Med.* **4**, 24 (2021). 33580109 10.1038/s41746-021-00394-8 PMC7881135

[9] Zhang, X. S., Tang, F., Dodge, H. H., Zhou, J. & Wang, F. MetaPred: meta-learning for clinical risk prediction with limited patient electronic health records. In Proc*.**25th ACM SIGKDD International Conference on Knowledge Discovery & Data Mining* 2487–2495 (Association for Computing Machinery, 2019). 10.1145/3292500.3330779 PMC8046258 33859865

[10] Hoffman, H. et al. Machine learning for clinical outcome prediction in cerebrovascular and endovascular neurosurgery: systematic review and meta-analysis. *J Neurointerv Surg*. 10.1136/jnis-2024-021759 (2024). 10.1136/jnis-2024-021759 38772570

[11] Kavakiotis, I. et al. Machine learning and data mining methods in diabetes research. *Comput. Struct. Biotechnol. J.* **15**, 104–116 (2017). 28138367 10.1016/j.csbj.2016.12.005 PMC5257026

[12] Adamson, B. et al. Approach to machine learning for extraction of real-world data variables from electronic health records. *Front. Pharmacol.* **14**, 1180962 (2023). 10.3389/fphar.2023.1180962 PMC10541019 37781703

[13] Wiest, I. C. et al. Privacy-preserving large language models for structured medical information retrieval. *NPJ Digit Med.* **7**, 257 (2024). 39304709 10.1038/s41746-024-01233-2 PMC11415382

[14] Huang, J. et al. A critical assessment of using ChatGPT for extracting structured data from clinical notes. *NPJ Digit Med.* **7**, 106 (2024). 38693429 10.1038/s41746-024-01079-8 PMC11063058

[15] Wiest, I. C. et al. Deep sight: enhancing periprocedural adverse event recording in endoscopy by structuring text documentation with privacy-preserving large language models. *iGIE* **3**, 447–452.e445 (2024). 10.1016/j.igie.2024.08.001 PMC12850884 41647922

[16] Rajkomar, A. et al. Scalable and accurate deep learning with electronic health records. *NPJ Digit Med.* **1**, 18 (2018). 31304302 10.1038/s41746-018-0029-1 PMC6550175

[17] Saito, T. & Rehmsmeier, M. The precision-recall plot is more informative than the ROC plot when evaluating binary classifiers on imbalanced datasets. *PLoS ONE* **10**, e0118432 (2015). 25738806 10.1371/journal.pone.0118432 PMC4349800

[18] Efron, B. *CBMS-NSF Regional Conference Series in Applied Mathematics* (SIAM, 1982).

[19] Lundberg, S. M. & Lee, S.-I. A unified approach to interpreting model predictions. In *Proc. 31st International Conference on Neural Information Processing Systems* 4768–4777 (Curran Associates Inc., 2017).

[20] Rädsch, T. et al. What your radiologist might be missing: using machine learning to identify mislabeled instances of X-ray images. In *Proc. 54th Hawaii International Conference on System Sciences* 1294 (2021).

[21] Zhao, J., Henriksson, A., Asker, L. & Boström, H. Predictive modeling of structured electronic health records for adverse drug event detection. *BMC Med. Inf. Decis. Mak.* **15**, S1 (2015). 10.1186/1472-6947-15-S4-S1 PMC4660129 26606038

[22] Ribeiro, M. T., Singh, S. & Guestrin, C. Why should I trust you?: explaining the predictions of any classifier. In *Proc. 22nd ACM SIGKDD International Conference on Knowledge Discovery and Data Mining* 1135–1144 (Association for Computing Machinery, 2016).

[23] McKinney, W. Data structures for statistical computing in Python. In *Proc. 9th Python in Science Conference* (eds. van der Walt S. & Millman J.) 56–61 (2010).

[24] Pedregosa, F. et al. Scikit-learn: machine learning in Python. *J. Mach. Learn. Res.* **12**, 2825–2830 (2011).

[25] Ke, G. et al. LightGBM: A highly efficient gradient boosting decision tree. In *Proc. 31st International Conference on Neural Information Processing Systems* 3149–3157 (Curran Associates Inc., 2017).

[26] Prokhorenkova, L., Gusev, G., Vorobev, A., Dorogush, A. V. & Gulin, A. CatBoost: unbiased boosting with categorical features. In *Proc. 32nd International Conference on Neural Information Processing Systems* 6639–6649 (Curran Associates Inc., 2018).

[27] Arik, S. Ö. & Pfister, T. Tabnet: attentive interpretable tabular learning. In *Proc. AAAI Conference on Artificial Intelligence* Vol. 35 6679–6687 (2021).

[28] Akritas M. G., Politis D. N. *Recent Advances and Trends in Nonparametric Statistics* (JAI Press, 2003).

[29] Sofaer, H. R., Hoeting, J. A., Jarnevich, C. S. & McPherson, J. The area under the precision-recall curve as a performance metric for rare binary events. *MEE* **10**, 565–577 (2019).

