# Supplementary material for: How machine learning on real world clinical data improves adverse event recording for endoscopy
Source: NPJ Digit Med. 2025 Jul 10;8:424. doi: 10.1038/s41746-025-01826-5 (PMC12246240; doi:10.1038/s41746-025-01826-5)
Supplement: Supplementary file 1 — Paper_Metadaten_ML_supplementary [file 41746_2025_1826_MOESM1_ESM.pdf]

Supplementary information

Consensus definitions for adverse events, including bleeding, perforation, and readmission

Bleeding[15] is defined as any major bleeding mentioned in the report that required intervention. If only coagulation or argon plasma coagulation was mentioned and no further reference to significant bleeding was made, it was not counted as a bleeding AE because this method could have been used prophylactically after polypectomy and minor bleeding. If an area was described as "spontaneously bleeding," it was counted as a bleeding AE. If the area examined already contained (old) blood on entry, this was not counted as a bleeding AE because it was interpreted as a source of bleeding other than from the procedure. If no major bleeding was described but the words "the bleeding has stopped" indicated that bleeding had occurred, we counted this as a bleeding AE. If bleeding becomes apparent after readmission, it was classified as adverse event readmission instead.

Perforation[15] is defined as any injury to the bowel wall up to the lamina muscularis propria. If a clip was used for "wall adaptation" and no further information was provided, this was not considered a result of perforation because it was unclear which bowel layers were adapted. If perforation becomes apparent after readmission, it was classified as adverse event readmission instead.

Readmission is defined as a hospital readmission within 30 days of discharge, with at least one adverse event related to colonoscopy with endoscopic mucosal resection (EMR) occurring during the initial hospital stay. Re-presentation to the emergency room without admission to any station was also counted as readmission. If bleeding or perforation became apparent after readmission, it was classified as an adverse event readmission.

Type of adverse event	Number of occurrences
Bleeding	36
Readmission	5
Perforation	4
Post polypectomy syndrome	1
Vomiting	1

Supplementary Table 1: Adverse events found in 200 randomly selected cases without any documented adverse event.

We analysed 200 randomly selected cases from our dataset at the University Hospital Mannheim that had no systematically documented adverse events. The type and number of adverse events within this selection is displayed in the above table. The analysis included endoscopy reports, discharge letters, readmission notes, and any other available data sources to identify undocumented adverse events. The search was not limited to complications bleeding, readmission and perforation but open to any adverse event. It can be argued that events such as readmission, perforation, and post-polypectomy syndrome are significant and should be systematically documented to ensure higher data quality. Bleeding, on the other hand, may not always be recorded, as it is often considered a minor adverse event that can be addressed immediately and may not require extensive follow-up. In our analysis of 200 cases without systematically documented adverse events, we identified 47 adverse events. When excluding bleeding (since minor bleeding events may be inconsistently documented) there were still 11 adverse events in this subset. Extrapolating this rate to the full set of 2,290 cases without central documentation of adverse events suggests that approximately 126 adverse events (excluding bleeding) may have gone unrecorded. This contrasts with the 194 centrally documented adverse events, indicating that roughly 40% of relevant events are currently missed, potentially even more when bleeding is included. In summary, this analysis provides evidence that some adverse events, even significant ones, remain undocumented.

For the machine learning part, we focused on the most common adverse events: bleeding, readmission and perforation. It is important to note that the aforementioned data includes only cases without documented adverse events, whereas the dataset used to train the machine learning model encompasses all cases, including those with documented adverse events.

Feature	Description
Charlson comorbidity index	A weighted index used to predict mortality by accounting for the presence of various comorbid conditions.
Age	Age of patient
Elective procedure	Indicates whether procedure was planned (elective) or urgent
Number of procedures	Total count of endoscopic procedures underwent during the hospitalization.
Barthel index	A measure of a patient's ability to perform daily activities, assessing functional independence.
Patient Clinical Complexity Level	A metric used to gauge the severity and complexity of a patient’s clinical condition.
Number of blood transfusions	Total count of blood transfusions administered during the hospital stay.
Admission-to-discharge time	The duration between the patient's admission and discharge.
Procedure-to-discharge time	Time elapsed between endoscopic procedure and the patient's discharge.
Procedure duration	The length of time taken for corresponding endoscopic procedure.
ICD-code on admission	ICD-code recorded as reason for admission
ICD-codes	A list of ICD-codes recorded during patient’s hospital stay
DRG-codes	Diagnosis-Related Group codes used to classify hospitalization costs and reimbursements based on diagnosis and treatment
Material used in endoscopy	Describes the specific materials and their number utilized during endoscopic procedures.
OPS-codes	Procedure codes from the Operation and Procedure Classification System (OPS), indicating surgeries or procedures performed

Supplementary Table 2: Metadata features used for adverse events type perforation and bleeding**.**

The features ICD-code on admission, ICD-codes, DRG-codes, Material used in endsocpy and OPS-codes were one hot encoded.

Feature	Description
Discharge to readmission time	Time between the patient's discharge and their next hospital admission.
ICD-codes at readmission	ICD codes listed during patients next hospital stay

Supplementary Table 3: Metadata features used for adverse events type readmission.

The feature ICD-codes at readmission was one hot encoded.

Urgency	Number of entries	Insurance type	Number of entries	Type of endoscopy	Number of entries
Elective	1492	Statutory health insurance	1204	Colonoscopy	2263
Emergency	4	Private	413	Sigmoidoscopy	234

Supplementary Table 4: Urgency, insurance type and type of endoscopy

Within the cohort (restricted to documented cases), most procedures were classified as elective, while only a minority (four cases) were classified as emergencies. The majority of patients were insured through the German statutory health insurance scheme. Significantly more colonoscopies were performed compared to sigmoidoscopies within the cohort.

Main diagnosis (ICD)	Description	Number of entries	Secondary diagnosis (ICD)	Description	Number of entries
K63.5	Polyp of the colon	400	K63.5	Polyp of the colon	23
D12.2	Benign neoplasm: Ascending colon	335	D12.6	Benign neoplasm: Colon, unspecified	6
D12.0	Benign neoplasm: Cecum	298	D12.0	Benign neoplasm: Cecum	5
D12.8	Benign neoplasm: Rectum	178	K92.2	Gastrointestinal bleeding, unspecified	4
D12.3	Benign neoplasm: Transverse colon	148	D12.3	Benign neoplasm: Transverse colon	4

Supplementary Table 5: Top 5 main and secondary diagnosis within the patient cohort.

The most common main diagnosis of the cohort were polyp of the colon or benign neoplasm within the colon, cecum or rectum.

Admission diagnosis (ICD)	Description	Number of entries	Admitting department	Number of entries
K63.5	Polyp of the colon	648	Surgical	1566
D12.6	Benign neoplasm: Colon, unspecified	329	Gastro-enterology	681
D12.2	Benign neoplasm: Ascending colon	142	Oncology	63
D12.0	Benign neoplasm: Cecum	122	Cardiology	57
D12.8	Benign neoplasm: Rectum	102	Emergency department	52

Supplementary Table 6: Top 5 admission diagnosis and admitting department.

The most common diagnoses were polyps of the colon and benign neoplasms within the colon, cecum, or rectum. The departments with the most admitted patients were the surgical and gastroenterology departments.

Documentation of adverse events	Number of entries
No documented adverse events	2296
Documented adverse events	194

Supplementary Table 7: Systematically documented adverse events.

Within the cohort, 194 adverse events were systematically recorded, while 2,296 cases had no such recorded events.

	Perforation manual labels	Perforation LLM labels	Bleeding manual labels	Bleeding LLM labels
AUC-ROC	0.863 [0.859 to 0.866]	0.844 [0.840 to 0.848]	0.834 [0.831 to 0.835]	0.829 [0.827 to 0.831]
AUC-PR	0.568 [0.561 to 0.574]	0.57 [0.563 to 0.577]	0.625 [0.621 to 0.629]	0.613 [0.608 to 0.617]

Supplementary Table 8: Comparison of model performance of manual vs. LLM-Generated Labels

We analyzed a subset of 500 samples for which both manual and LLM-generated labels were available, performing 1,000 bootstrapping iterations to assess potential differences in model performance for adverse events perforation and bleeding. The machine learning algorithm was trained twice: once using manual labels and once using LLM-generated labels. Both versions were evaluated against the ground truth, defined as the manual labels. In each iteration, training data was created through bootstrapping, and evaluation was performed on the out-of-bag samples. The average values of AUC-ROC and AUC-PR are reported, along with 95% confidence intervals in brackets. As shown, the AUC-ROC and AUC-PR values for manual and LLM-generated labels are roughly similar, indicating that the model achieves comparable performance when trained on either manual or LLM-generated labels. Strictly speaking, this observation is limited to the 500 cases used in this analysis, but it suggests that similar trends may extend to the full dataset of 2,490 cases.

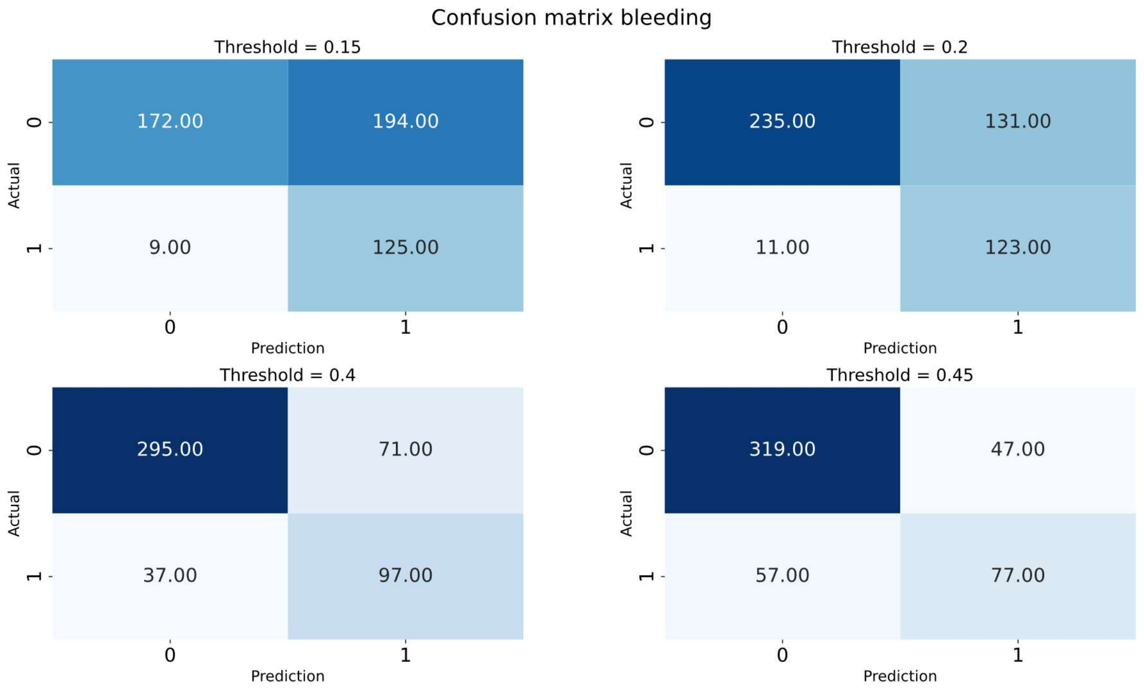

Supplementary Figure 1: Confusion matrix for bleeding

Confusion matrix for adverse event bleeding for different classification thresholds. The model was tested on 500 manual labels representing the ground truth.

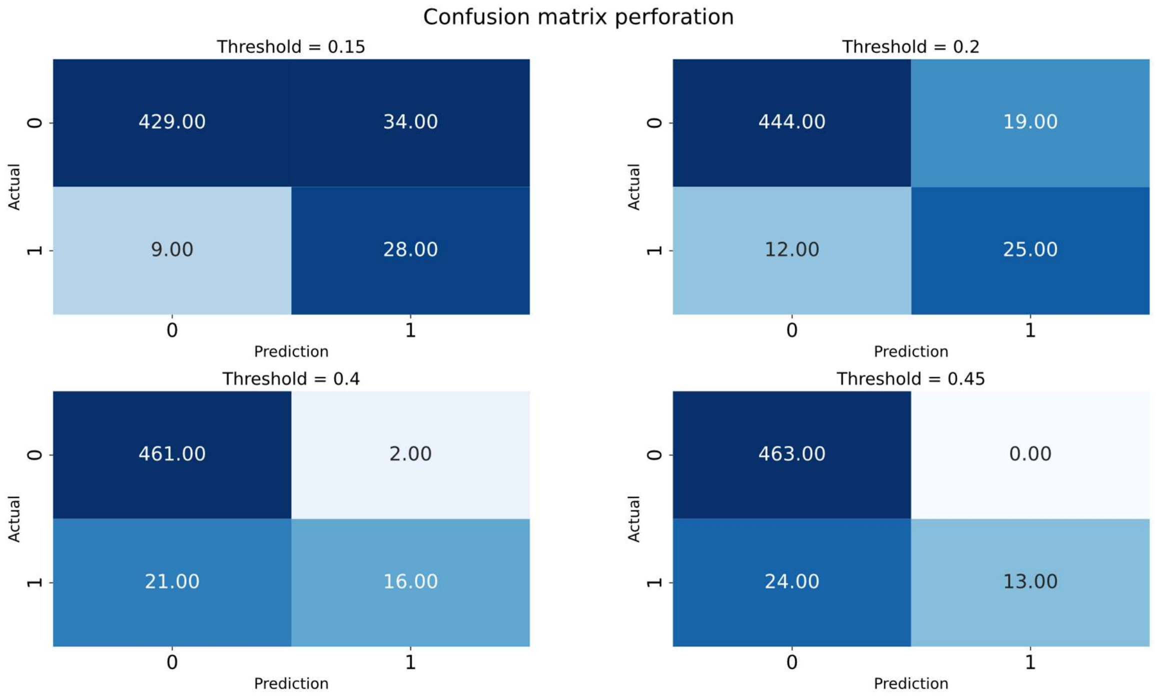

Supplementary Figure 2: Confusion matrix for perforation

Confusion matrix for adverse event bleeding for different classification thresholds. The model was tested on 500 manual labels

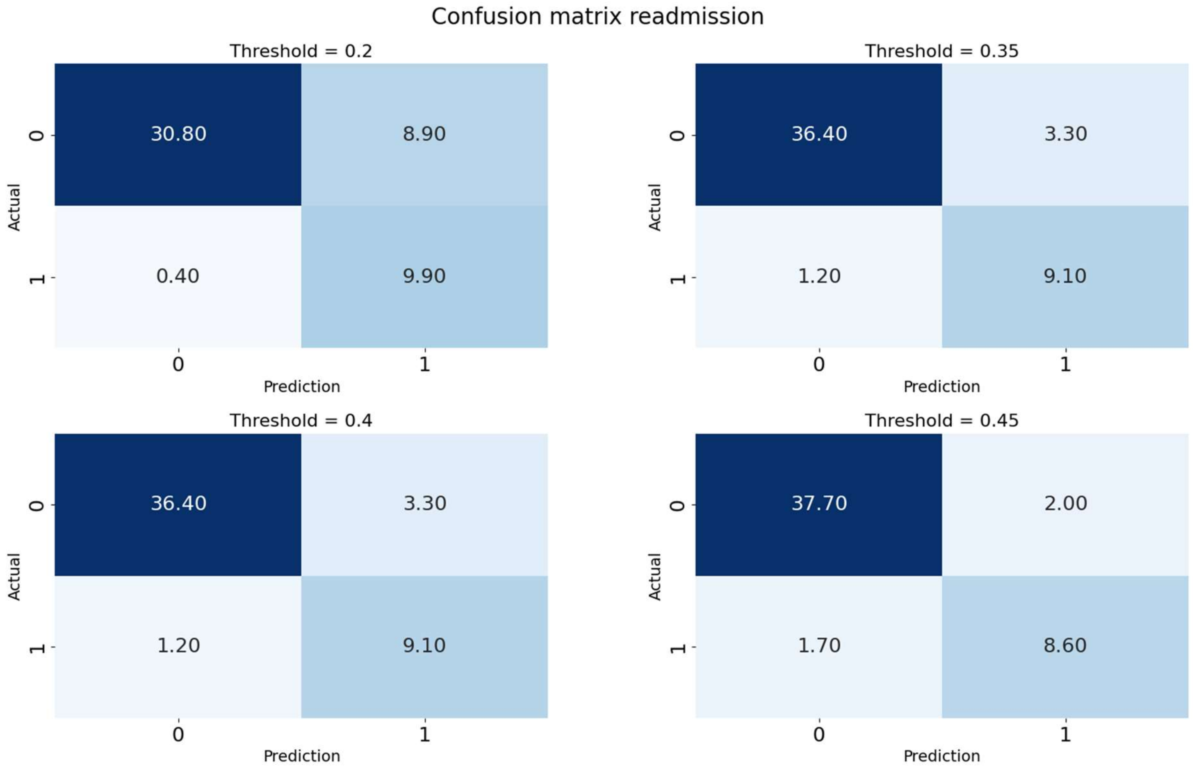

Supplementary Figure 3: Confusion matrix for readmission

Confusion matrix for adverse event readmission for four classification thresholds. The model was tested on 50 test cases. The results are averaged over 100 runs using random subsampling each time selecting different cases for the training and test data set. The fractional values result from averaging; however, the total sum remains 50 test cases.

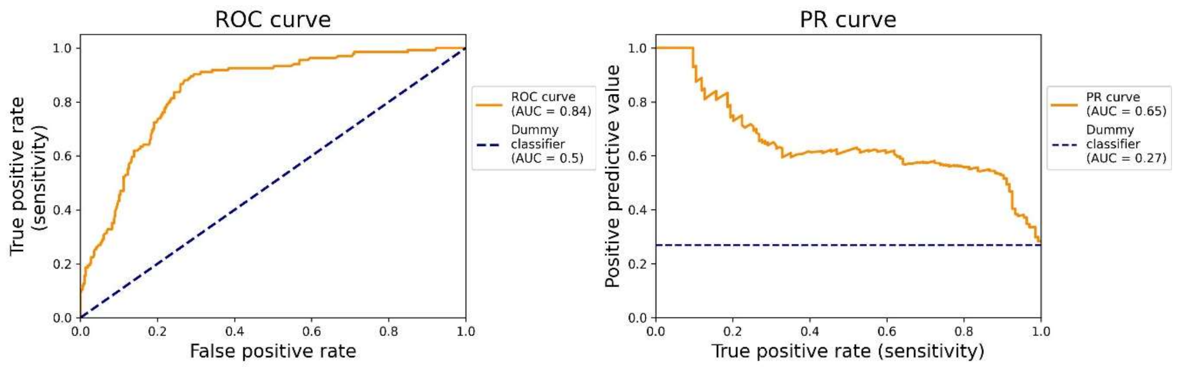

Supplementary Figure 4: ROC- und PR-curves for bleeding

The ROC- and PR-curves for adverse event bleeding were obtained by testing on 500 cases with manual labels

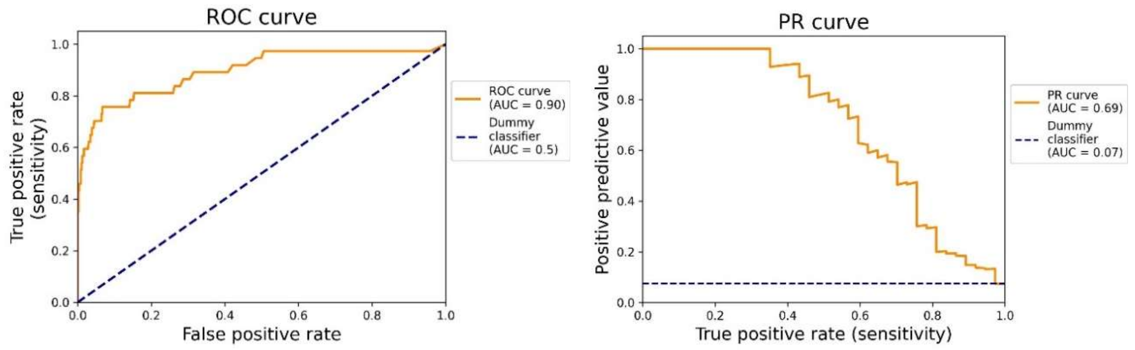

Supplementary Figure 5: ROC-and PR-curves for perforation

The ROC- and PR-curves for adverse event perforation were obtained by testing on 500 cases with manual labels

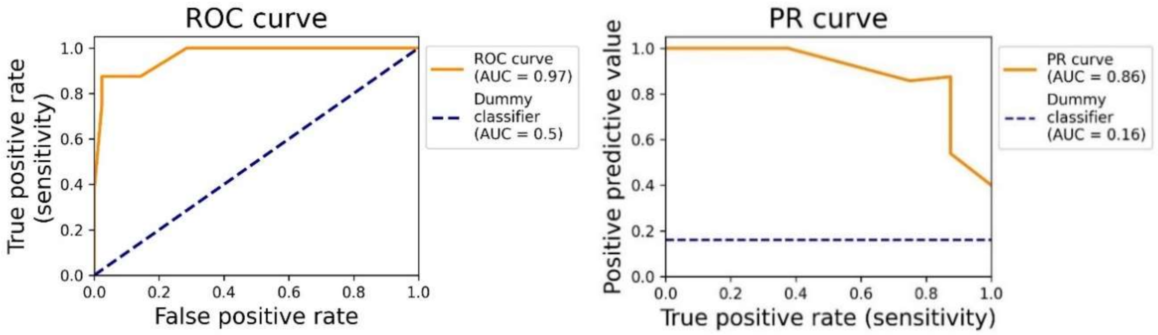

Supplementary Figure 6: ROC- and PR-curves for readmission

The ROC- and PR-curves for adverse event readmission were obtained by testing on 50 cases with manual labels. A snapshot from a single run is displayed.

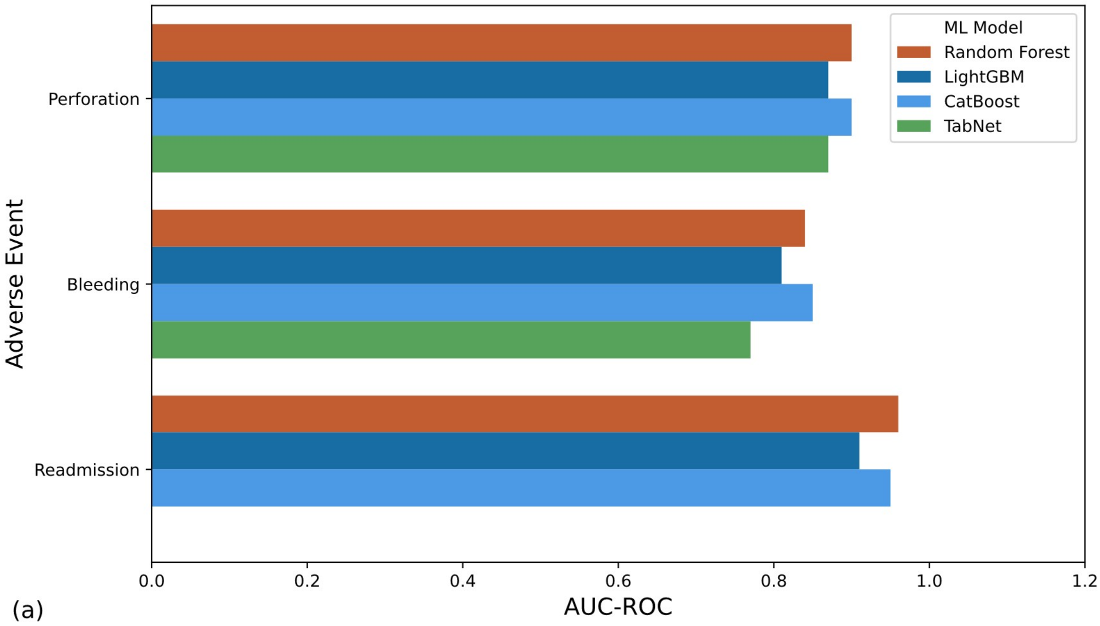

(a)

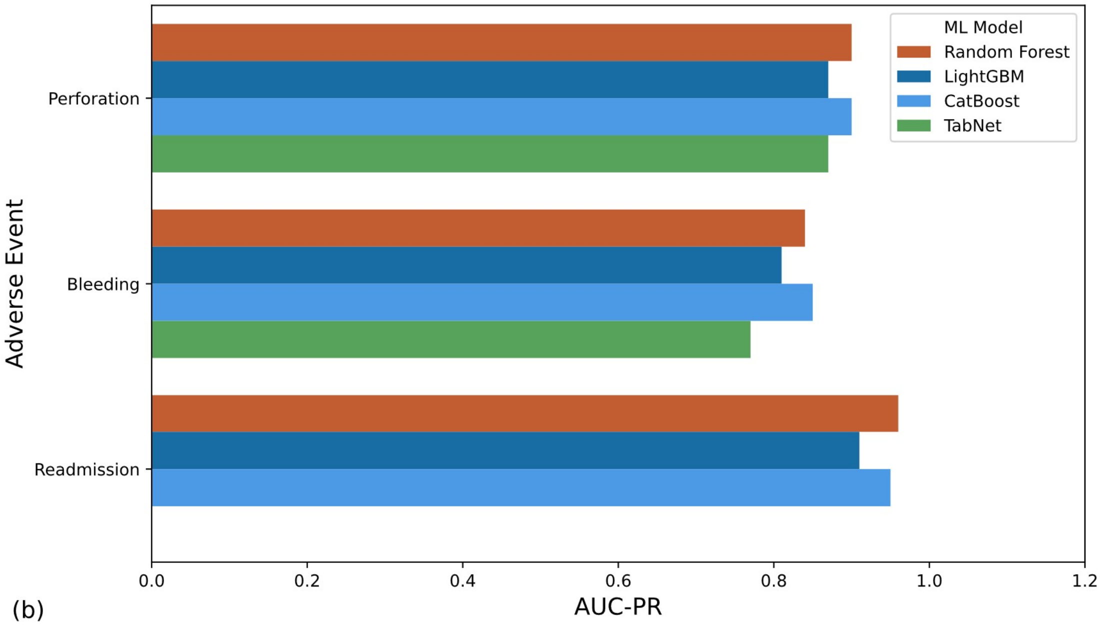

(b)

Supplementary Figure 7: Benchmarking model performance.

AUC-ROC (a) and AUC-PR (b) for machine learning models LightGBM, CatBoost and TabNet tested on adverse events perforation, bleeding and readmission are displayed. The results are compared to the Random Forest used in this work. Random forest and CatBoost demonstrated the best performance, with CatBoost on par with random forest. In contrast, LightGBM and TabNet achieved lower scores. Following implementation choices were made:

LightGBM underwent hyperparameter tuning using five-fold cross-validation on the training data to reduce overfitting.

CatBoost was run using 500 features and did not require hyperparameter tuning.

TabNet was also run with 500 features and is designed to require minimal hyperparameter tuning[27]. To mitigate overfitting, early stopping based on AUC-PR on a validation set was employed. Due to the small sample size, TabNet was not tested on adverse event readmission.

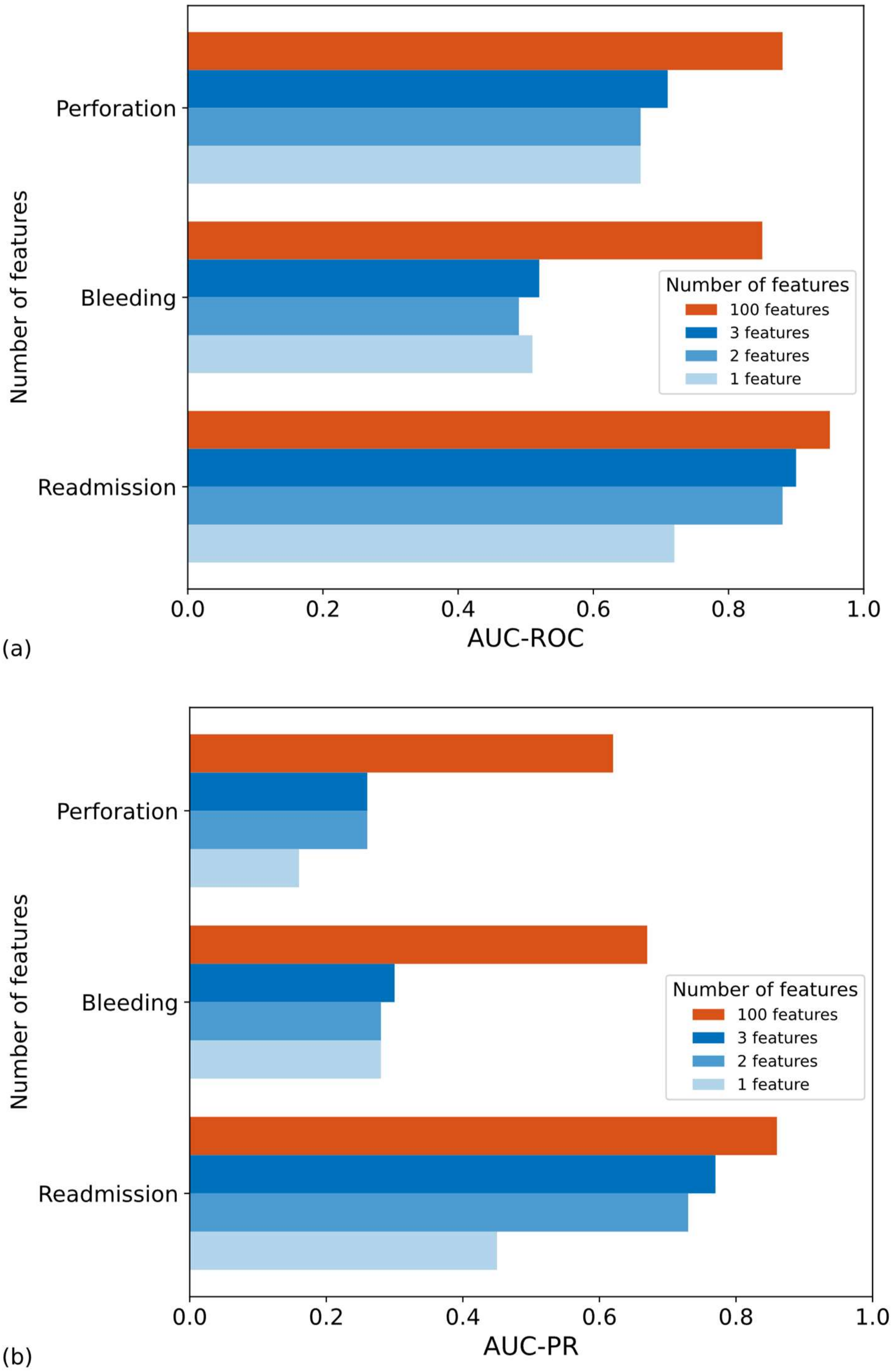

Supplementary Figure 8: AUC-ROC and AUC-PR for 1,2,3 and 100 features.

The algorithm was run with 1,2,3 and 100 features. The features were selected through backward elimination. A decline in performance, measured by AUC-ROC (shown in panel **(a)**) and AUC-PR (shown in panel **(b)**), is observed as the number of features decreases. This decline is more pronounced for adverse events perforation and bleeding. In the case of adverse events in connection to readmission it is noticeable but less severe.

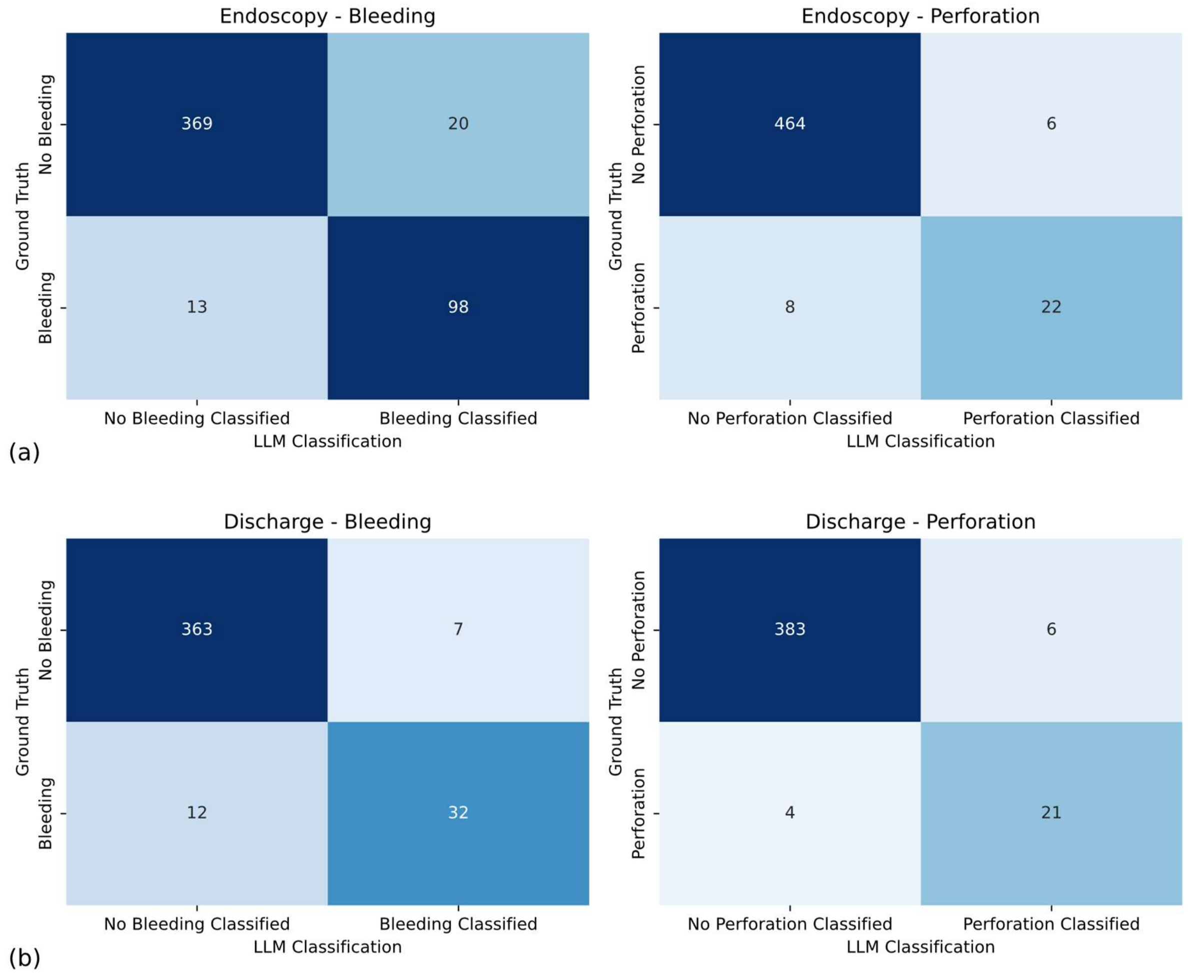

Supplementary Figure 9: Comparison of large language models labels and ground truth (given by manual labels).

The labels generated by the LLM model are compared to the corresponding available manual labels (in total 500 cases) representing the ground truth. The analysis considered the endoscopy reports (shown in panel **(a)**) and discharge letters (shown in panel **(b)**) separately for adverse events bleeding and perforation. A total of 500 endoscopy reports with manual labels were available. However, only 414 of these reports were accompanied by a discharge letter. For training and testing the random forest machine learning model, the labels obtained from the endoscopy reports and discharge letters were combined. For endoscopy reports, bleeding reached a sensitivity of 0.88, specificity of 0.95, and a positive predictive value of 0.83. For perforations, the endoscopy report showed a sensitivity of 0.76, specificity of 0.99, and a positive predictive value of 0.79. When examining the discharge letter, the sensitivity for bleeding was 0.73, specificity was 0.98, and the positive predictive value was 0.82. For perforations in the discharge letter, sensitivity increased to 0.84, specificity remained high at 0.98, and the positive predictive value was 0.78.
